# Alcohol use disorder is associated with DNA methylation-based shortening of telomere length and regulated by *TESPA1*: implications for aging

**DOI:** 10.1038/s41380-022-01624-5

**Published:** 2022-06-15

**Authors:** Jeesun Jung, Daniel L. McCartney, Josephin Wagner, Daniel B. Rosoff, Melanie Schwandt, Hui Sun, Corinde E. Wiers, Luana Martins de Carvalho, Nora D. Volkow, Rosie M. Walker, Archie Campbell, David J. Porteous, Andrew M. McIntosh, Riccardo E. Marioni, Steve Horvath, Kathryn L. Evans, Falk W. Lohoff

**Affiliations:** 1 Section on Clinical Genomics and Experimental Therapeutics, National Institute on Alcohol Abuse and Alcoholism, National Institutes of Health, Bethesda, MD USA; 2 Centre for Genomic and Experimental Medicine, Institute of Genetics and Cancer, University of Edinburgh, Edinburgh, UK; 3 Office of the Clinical Director, National Institute on Alcohol Abuse and Alcoholism, National Institutes of Health, Bethesda, MD USA; 4 Laboratory of Neuroimaging, National Institute on Alcohol Abuse and Alcoholism, National Institutes of Health, Bethesda, MD USA; 5 Department of Biostatistics, Fielding School of Public Health, University of California Los Angeles, Los Angeles, CA USA; 6 Department of Human Genetics, David Geffen School of Medicine, University of California Los Angeles, Los Angeles, CA USA

**Keywords:** Predictive markers, Molecular biology

## Abstract

Chronic heavy alcohol consumption is associated with increased mortality and morbidity and often leads to premature aging; however, the mechanisms of alcohol-associated cellular aging are not well understood. In this study, we used DNA methylation derived telomere length (DNAmTL) as a novel approach to investigate the role of alcohol use on the aging process. DNAmTL was estimated by 140 cytosine phosphate guanines (CpG) sites in 372 individuals with alcohol use disorder (AUD) and 243 healthy controls (HC) and assessed using various endophenotypes and clinical biomarkers. Validation in an independent sample of DNAmTL on alcohol consumption was performed (*N* = 4219). Exploratory genome-wide association studies (GWAS) on DNAmTL were also performed to identify genetic variants contributing to DNAmTL shortening. Top GWAS findings were analyzed using *in-silico* expression quantitative trait loci analyses and related to structural MRI hippocampus volumes of individuals with AUD. DNAmTL was 0.11-kilobases shorter per year in AUD compared to HC after adjustment for age, sex, race, and blood cell composition (*p* = 4.0 × 10^−12^). This association was partially attenuated but remained significant after additionally adjusting for BMI, and smoking status (0.06 kilobases shorter per year, *p* = 0.002). DNAmTL shortening was strongly associated with chronic heavy alcohol use (*p*s < 0.001), elevated gamma-glutamyl transferase (GGT), and aspartate aminotransferase (AST) (*ps* < 0.004). Comparison of DNAmTL with PCR-based methods of assessing TL revealed positive correlations (*R* = 0.3, *p* = 2.2 × 10^−5^), highlighting the accuracy of DNAmTL as a biomarker. The GWAS meta-analysis identified a single nucleotide polymorphism (SNP), rs4374022 and 18 imputed ones in Thymocyte Expressed, Positive Selection Associated 1(*TESPA1*), at the genome-wide level (*p* = 3.75 × 10^−8^). The allele C of rs4374022 was associated with DNAmTL shortening, lower hippocampus volume (*p* < 0.01), and decreased mRNA expression in hippocampus tissue (*p* = 0.04). Our study demonstrates DNAmTL-related aging acceleration in AUD and suggests a functional role for *TESPA1* in regulating DNAmTL length, possibly via the immune system with subsequent biological effects on brain regions negatively affected by alcohol and implicated in aging.

## Introduction

Chronic heavy alcohol consumption reduces life expectancy, leads to premature aging worldwide [1–3], and is associated with age-related diseases such as cardiometabolic diseases, cancer and decline in brain function [4–10]. It has been reported that individuals with heavier alcohol use among those with alcohol use disorder (AUD) have more pronounced age-related disease and accelerated biological aging compared to healthy individuals [11, 12]. Even though AUD and heavy alcohol consumption have been shown to be risk factors for premature aging, the underlying mechanisms between alcohol use and the aging process have not been well studied [13, 14].

An emerging biomarker of cellular aging is telomere length (TL), which has been associated with hypertension, diabetes, serious mental illnesses, and early mortality [5, 14–16]. Telomeres are tandem repetitive nucleotide sequences at chromosome ends that play a critical role in facilitating complete chromosome replication. Telomeres protect genomic DNA against double-strand breaks and DNA end-joining and recombination. As a normal cellular process, a small portion of telomeric DNA is lost with each cell division [17]. TL shortens with natural aging because it is not fully replicated during every cell division, so TL has served as a biological marker to predict lifespan [18]. TL is influenced by genetics with a heritability of 34–82% [19–22], and is associated with lifestyle factors, including cumulative stress exposures, smoking, alcohol use, and psychiatric disorders that may affect cell aging and induce damage to DNA [17, 23].

Although cellular TL shortening has been linked with AUD and other substance use disorders, the association with broader alcohol consumption phenotypes remains unclear [23–26]. In addition, despite the importance of TL as a biomarker to illustrate a direct or indirect association with age-related traits and health complications, measuring TL remains challenging and subject to confounding factors, including DNA extraction methods and other technical variations [27].

In contrast to the classical approaches to measure TL, innovative methods utilizing DNA methylation are now available to predict TL [28]. Genome-wide DNA methylation (DNAm) profiling has been used in AUD populations [29, 30] and can be used to predict cellular DNA methylation telomere length (DNAmTL) [28]. In addition, DNAmTL is easier to measure compared to traditional polymer chain reaction (PCR)-based TL assessments and might be a more reliable biomarker tracking clinically important traits because methylation signatures might represent changes in transcriptional programs and reflect cumulative effects of exposures over time [28, 31, 32]. Moreover, DNAmTL correlates strongly with age and performs better in predicting lifespan, and also exhibits strong negative correlations with other epigenetic clocks, with proxied TL reduction corresponding to epigenetic age acceleration [28]. Similarly, DNAmTL is heritable (h2 ≈ 0.48) and differs across race and ethnicities [28]; however, little is known about the genetic architecture underlying DNAmTL.

In this study, we first aimed to investigate the relationship between AUD and DNAmTL shortening in the largest AUD cohort currently available. Furthermore, we investigated whether elevated liver function enzyme (LFE) biomarkers, as indicators of AUD severity, were associated with DNAmTL. Replication was performed in a large sample with a focus on alcohol consumption. Technical validity of DNAmTL was conducted by comparing DNAmTL to classic PCR-based methods. Given that aging is often associated with cognitive decline, memory loss and decreased hippocampal volumes, we also explored correlation of DNAmTL with hippocampal structure. The secondary aims of our study were to perform a genome-wide association study (GWAS) on DNAmTL to identify genetic contributions to shortening in DNAmTL, and to conduct cis-expression quantitative trait loci (eQTL) analyses of top targets to explore biological relevance.

## Materials and methods

### Study participants

The sample consisted of 615 participants, 372 with AUD and 243 healthy controls (HC) recruited at the National Institute on Alcohol Abuse and Alcoholism (NIAAA) at the National Institutes of Health (NIH), USA. All study participants completed the Structured Clinical Interview for Diagnostic and Statistical Manual of Mental Disorders (DSM)-IV-TR (SCID-IV) to determine an alcohol-dependence (AD)/AUD diagnosis. Given the overlap between the DSM-IV alcohol dependence criteria and the DSM-5 AUD criteria, all participants with AD also met criteria for AUD diagnosis. Participants provided a blood sample that was used for genome-wide DNA methylation analysis as well as genome-wide genotyping analysis and clinical biomarker collection, including LFE for gamma-glutamyl transferase (GGT), alanine aminotransferase (ALT), and aspartate aminotransferase (AST). Participants also completed self-report questionnaires, including the Timeline Followback (TLFB) [33], a measure of alcohol intake over the previous 90 days, and the Fagerström Test for Nicotine Dependence (FTND) [34]. FTND scores range from 0 to 10 with participants scoring 0 indicating non-smokers, and those with scores >0 defined as smokers. All study participants provided written informed consent in accordance with the Declaration of Helsinki and were compensated for their time. The study was approved by the Institutional Review Board of the NIH.

### DNA methylation measurements

DNA methylation levels from whole blood samples were assessed using an Infinium MethylationEPIC BeadChip microarray (Illumina Inc., San Diego, California). We implemented a robust strategy to minimize the potential confounding influence of batch effects on the hypotheses tested in the data set. Prior to sample processing, all major potential sources of batch-based confounding were considered including sodium bisulfite modification batch (*N* = 96/plate) and microarray batch (*N* = 8/array). Within technical batches, experimental conditions as well as covariates were matched such that distributions in age, sex, race, and smoking status did not vary between primary experimental groups within a batch. As such, even with the presence of technical batch effects, the statistical interpretation of findings would remain unaffected. Following sample processing and array hybridization, we performed a batch correction using scale-based correction of type I and type II probes followed by using Minfi to correct background noise and WateRmelon for individual red and green channel quantile normalization prior to beta calculation using the Dasen method. Evaluation of red and green channel and beta frequency distributions between batches did not reveal significant cross batch effects and no further statistical manipulation of the data was deemed necessary. After methylated and unmethylated intensities were quantile-normalized, *β*-values were calculated using the ratio of intensities between methylated and unmethylated alleles. Relative proportions of 6 types of cells (granulocytes, monocytes, natural killer cells, B cells, CD4 + T cells, and CD8 + T cells) were estimated using the Houseman estimation [35]. The final methylome dataset consisted of *β*-values for 835,928 CpG sites for all 615 participants.

### Calculating DNAm telomere length and statistical analysis

We estimated DNAm telomere length (DNAmTL) using a previously published weighted average of 140 CpG sites selected by regressing measured leukocyte telomere length (LTL—assessed by terminal restriction fragmentation) on blood methylation data of 2256 individuals [28]. The predicted LTL, DNAmTL, possesses the same units (kilobase per year) as that of mean terminal restriction fragments (TRF) measured by Southern blotting [28] and the estimated DNAmTL in our sample has the same unit. Age-adjusted DNAmTL was calculated by regressing DNAmTL on chronological age, with a negative value suggesting that TL is shorter than expected at a given age whereas a positive value suggests longer than expected [36–39]. DNAmTL measures have been shown to significantly correlate with LTL measured with TRF in 12 independent cohorts (*R* = 0.4–0.5), indicating that it is an accurate alternative [28]. To validate the performance of DNAmTL in our sample, we also examined the correlation between DNAmTL and LTL measured by a ratio of the telomere (T) to albumin (S) ratio (T/S ratio) in 191 HC that did not meet DSM-IV alcohol dependence criteria.

To investigate differences in age-adjusted DNAmTL between AUD and HC and associations with alcohol consumption in the AUD cohort (i.e. number of heavy drinking days in a past 90 days), a linear model with adjustment of age, sex, race, AUD diagnosis, and estimated proportions of six blood cell types was used (basic model). The alcohol consumption variables were standardized (mean = 0, SD = 1) to improve interpretation of results from a different scale. The fully adjusted model included additional covariates for smoking status and body mass index (BMI), which are potential confounding factors influencing the aging process (full model). Additional linear models, with the aforementioned basic and full covariate structures, examined if the standardized alcohol-associated biomarkers (i.e. GGT, ALT, AST) were associated with age-adjusted DNAmTL. Furthermore, additional adjustment for comorbid substance use disorders and psychiatric disorders was performed in the basic model. There was no evidence to suggest a non-Gaussian distribution of the residuals from the basic and full models (Shapiro-Wilk test, *p* > 0.05). As other drug use disorders may be associated with DNAmTL shortening, we conducted a sensitivity analysis by removing individuals with any comorbid drug dependences in AUD and compared the results to those with AUD with any comorbid substance use disorder. Statistical analyses were performed in R version 4.0.5 [40].

### GWAS analysis for DNAm telomere length

A genome-wide association study (GWAS) was performed in European Ancestry (EA) and African Ancestry (AA) participants separately with the Illumina OmniExpress and Illumina OmniExpressExome BeadChips (Illumina, San Diego, CA) and the imputed genotypes. The imputation of EA and AA was carried out separately by Minimac4 [41] using 1000 Genomes phase 3 panels as a reference sample [42]. To obtain ancestry/race information, we ran EIGENSTRAT [43] and conducted a population stratification analysis using the genome-wide data of all participants and 2504 individuals of the 1000 Genomes Project Phase 3.

Based on the genetically-identified EA and AA individuals with all imputed genotypes, we conducted a series of quality control (QC) procedures within each race group including: imputation accuracy (R2 > 0.7), sex check by X chromosome, Hardy-Weinberg equilibrium test in a control sample in EA and AA separately (*P* > 10^−5^), SNP genotyping rate (>0.99) and missingness by subjects (<0.03), and minor allele frequency (MAF) over 1%. The final sample size for the GWAS analyses after QC procedure was 297 in EA and 280 in AA. The total number of SNPs tested in the GWAS was 8,640,517 for EA and 14,892,985 for AA.

A linear regression model was utilized to test for association between each SNP and DNAmTL after adjusting for age, sex, smoking status, BMI, blood cell counts, and AUD diagnosis. All analyses were performed in EA and AA individuals separately with an additive genetic model. We then carried out a meta-analysis with a fixed-effect model weighted by inverse variance using the GWAS results of EA and AA samples using METAL [44]. SNPs that had substantial heterogeneity across the two GWAS results (Cochran’s Q test *p*-value <0.05 and heterogeneity index *I*^2^ > 75%) were removed. The total number of SNPs for the GWAS meta-analysis was 7,230,852. The threshold for the GWAS significant SNPs was set at *P* < 5 × 10^−8^. All genetic analyses were conducted using PLINK 1.90 software.

### In silico functional analyses and neuroimaging hippocampus volume data

Given that a SNP in *TESPA1* was a top finding in the meta GWAS and *TESPA1* is highly expressed in hippocampus and cerebral cortex regions (www.proteinatlas.org [45]), we performed a cis-expression quantitative trait loci (eQTL) analysis for *TESPA1* to examine correlation between the top variant and the transcript expression level in hippocampus tissue. We used the Brain Expression Quantitative Trait Loci (eQTL) Almanac (BRAINEAC) database [46], where the transcripts and exon-specific expression data were available across 10 brain regions from 134 neurologically normal European subjects.

### Structural neuroimaging of MRI

A subset of our AUD participants (*n* = 144) completed a structural magnetic resonance imaging (MRI) scan study. Hippocampus volumes were measured using the standard Freesurfer (version 5.3.0; surfer.nmr.mgh.harvard.edu) pipeline [47]. The individual T1-weighted images were automatically segmented to measure gray matter volume of structures [48] using the following steps: The images were resampled to 1 mm^3^ voxels and transformed to Talairach space; the intensity non-uniformity was corrected [49]; the skull was stripped from the images [50]; and finally auto-segmentation was performed with labels assigned based on probabilistic location of structures. We conducted a reliability test by examining a random number of the auto-segmented volumes from FreeSurfer’s QA Tools. This included checking for outliers, calculating signal-to-noise ratio, and visually examining generated snapshots of brain volume segmentation. Hippocampus volumes were analyzed using a linear model with the full model additional adjustment for estimated Total Intracranial Volume (eTIV).

### Replication studies with Generation Scotland (GS): Scottish Family Health Study

Generation Scotland (GS) is a family-structured, population-based cohort study of over 24,000 people from across Scotland, aged between 18 and 99 years at the study baseline (2006–2011). A broad set of phenotype data were collected at baseline and data linkage to electronic health records has enabled follow-up to collect information on incident disease outcomes. Full details have been reported previously [51, 52]. DNA was obtained from whole blood in ~20,000 people at the study baseline and alcohol consumption was measured for the week prior to sampling. Of the participants reporting normal levels of alcohol consumption relative to a typical week, 4219 also had DNA methylation data, processed in two sets. For set 1, a genetic relationship matrix was built using GCTA-GRM, and a relatedness coefficient of <0.025 was specified to exclude related individuals and we used only unrelated subject (*n* = 1501). Set 2 are unrelated (genetic relatedness <0.05) to each other, and to the participants from Set 1 (*n* = 2718). The quality control steps for Set 1 and Set 2 were nearly identical to one another and full details have been reported previously. More detailed descriptions are included in the Supplementary Methods. All statistical analyses were performed as per the discovery analyses, with additional adjustment of 20 genetic principal components.

## Results

Study participant characteristics are described in Supplementary Table S1. Participants with AUD and HC differed significantly in chronological age (*p* < 0.0001) and AUD cases were older with a mean age of 44.5 years (*SD* = 11). AUD cases had a higher proportion of males (*p* = 0.005) and smokers (*p* < 0.0001), but neither group differed in race/ancestry. Individuals with AUD had increased LFTs and heavier clinical alcohol consumptions than the HC group (*p* < 0.0001). The comorbidity rates of five illicit drug dependences (opioid, cocaine, cannabis etc.), MDD, and any anxiety disorder between AUD and HC were different (*p* < 0.01) (Supplementary Table S1). Importantly, we validated that DNAmTL was significantly correlated with LTL among 191 HC from our sample (*R* = 0.3, *p* = 2.2 × 10^−5^ in Fig. S1), which confirms the usefulness of DNAmTL as a proxy to study TL-related traits.

### Association of DNAm telomere length with clinical phenotypes

**Fig. 1:**
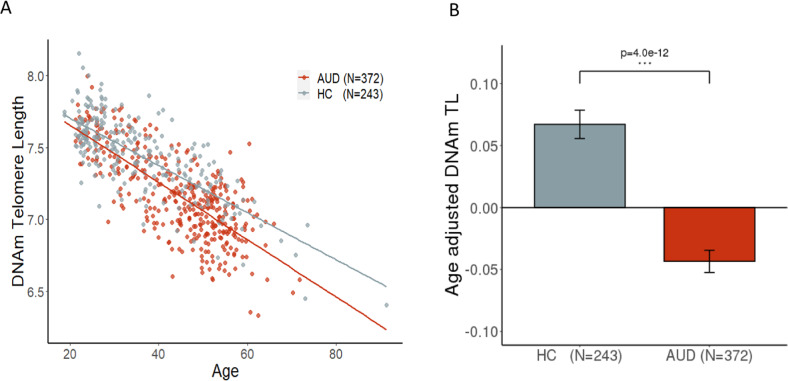
Association of age-adjusted DNAmTL with alcohol use disorder. **A** The scatter plot describes the DNAmTL with a linear fit at AUD and HC respectively. Individuals with AUD had rapid decrease in DNAmTL comparing to HC. **B** The bar plots show estimated means of the Age-adjusted DNAmTL after controlling for sex, race, six blood cell counts (basic model). The DNAmTL differed significantly between AUD cases and HC (*β* = −0.11 kilobases per year, *p* *=* 4.0 × 10^−12^).

DNAmTL decreased with age more rapidly in AUD than HC (Fig. 1A) and the age-adjusted DNAmTL was 0.11 kilobases/yr shorter in AUD cases compared to HC in the basic model (Fig. 1B, *β* = −0.11, *p* = 4.0 × 10^−12^). The decreased DNAmTL in AUD remained significant after additional adjustment for smoking status, BMI, and estimated blood cell proportions (*β* = −0.06, *p* = 0.002). Consistently, we found that DNAmTL was negatively associated with clinical measures of alcohol consumptions; DNAmTL was shortened by 0.04 kilobases/yr for every standard deviation (SD = 8.2) of drinks per day in the past 90-days (Table 1, *β* = −0.04; *p* = 2.1 × 10^−6^). The negative association remained significant in the full model (*β* = −0.03; *p* = 0.0002). Increased total drinks and heavy drinking days were also negatively correlated with DNAmTL (*β* = −0.04, ps < 0.001). The accelerated DNAmTL shortening was related to an increased number of drinking days and alcohol dependence scale (ADS) score only in the basic model (*p* < 0.05). Further analysis revealed that elevated GGT and AST were associated with shortened DNAmTL (*β* = −0.02; ps < 0.005) in both models even after adjusting for multiple testing. These negative associations were also present within AUD cases (Table S2). Sensitivity analysis showed that after removing all comorbid substance use disorders (SUD) among individuals with AUD, the results were attenuated, likely due to reduced sample size and phenotype variability. In contrast, individuals with AUD and comorbid SUD had stronger associations between DNAmTL and drinking variables/GGT suggesting that phenotype severity/heterogeneity contributes to shortened DNAmTL (Table S3). Sex-specific analysis exhibited that males had stronger association between DNAmTL shortening and alcohol use as well as liver function enzyme abnormalities, while females had associations of DNAmTL with mainly alcohol use (Table S4). Additional adjustment for comorbid psychiatric disorders such as either/both drug dependences and mood disorders did not change our main findings of the association between alcohol and DNAmTL shortening (Table S5). Furthermore, the replication study with the two datasets of Generation Scotland (GS) cohort showed that increased weekly alcohol use was associated with shortened DNAmTL (*β* = −0.002, *p* < 10^−5^, Table 1) in both models.

**Table 1:** Association of DNAmTL with alcohol use and liver function enzyme biomarkers.

	Basic model			Full model		
	*β*	*SE*	*P*-value	*β*	*SE*	*P*-value
**NIAAA sample**						
Total drinks	−0.042	0.008	**3.2** × **10**^**−7**^	−0.031	0.008	**0.0001**
No of drinking days	−0.023	0.010	**0.03**	−0.010	0.010	0.34
Average drinks per day	−0.040	0.008	**2.1** × **10**^**−8**^	−0.030	0.008	**0.0002**
Heavy drinking days	−0.039	0.011	**0.0002**	−0.022	0.011	**0.04**
ADS score	−0.003	0.001	**0.004**	−0.002	0.001	**0.023**
GGT	−0.020	0.007	**0.003**	−0.021	0.006	**0.002**
ALT	−0.008	0.007	0.25	−0.006	0.007	0.36
AST	−0.019	0.007	**0.005**	−0.018	0.007	**0.005**
**GS sample** of unit of weekly alcohol use						
Data set1 (*n* = 1501)	−0.0017	0.00032	**2.09** × **10**^**−7**^	−0.0009	0.00032	**0.003**
Data set2 (*n* = 2718)	−0.0018	0.00025	**1.44** × **10**^**−12**^	−0.0012	0.00025	**4.64** × **10**^**−6**^

*AUD* alcohol use disorder, *GGT* gamma-glutamyl transferase, *ALT* alanine aminotransferase, *AST* aspartate aminotransferase, *ADS* Alcohol Dependence Scale, Heavy drinking days are defined as ≥4 drinks a day for females; ≥5 drinks a day for males. Models were adjusted for age, gender, race, AUD diagnosis, six blood cell counts in the basic model, and additionally adjusted for smoking status, and body mass index in the full model, *NIAAA* National Institute on Alcohol Abuse and Alcoholism, *GS* Generation Scotland cohort.

Bold values indicate statistical significance *P* < 0.05.

### GWAS of age-adjusted DNAm telomere length

**Fig. 2:**
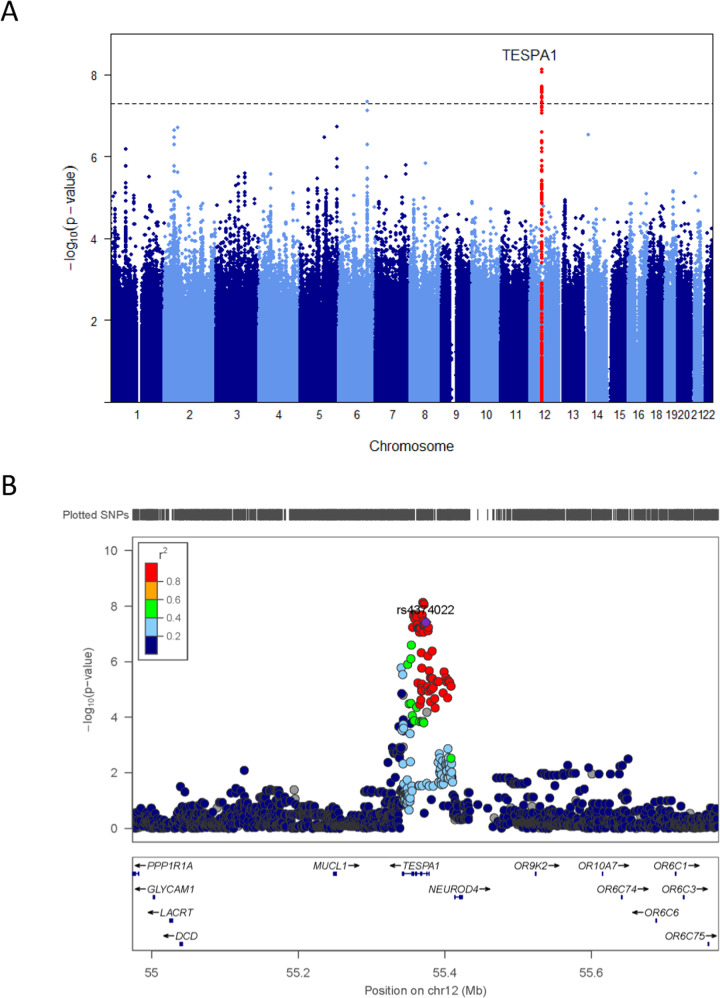
Cross-ancestry GWAS meta-analyses of DNAmTL identifies *TESPA1*. **A** Manhattan plot shows the meta-analysis *P*-values combining the results of EA and AA GWAS based on a fixed effect model using weight of inverse variance. EA and AA studies comprised of 297 EA and 280 AA individuals. The *y*-axis reports -log transformed Meta *P*-values. The horizontal dashed line corresponds to the genome-wide association threshold (*p* = 5.0 × 10^−8^). All SNPs in *TESPA1* were colored with red. **B** Regional association plot of *TESPA1* associated with DNAmTL shortening. The *y*-axis shows the -log-transformed meta-analysis *P*-value. The colors represent linkage disequilibrium (LD) R^2^ in European Ancestry Sample.

**Fig. 3:**
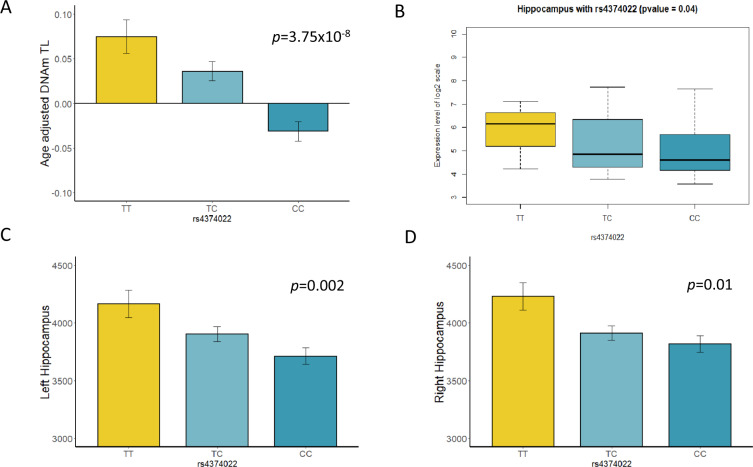
Association of *TESPA1* rs4374022 with hippocampus volumes and mRNA expression. **A** The bar plot shows estimated means of Age-adjusted DNAmTL adjusted for sex, race, blood cell proportions, BMI, and smoking status in all samples combined. Age-adjusted DNAmTL differed significantly between genotype groups (*p* = 3.75 × 10^−8^). Effect allele C of genome-wide significant SNP rs4374022 is associated with DNAmTL shortening by 0.058 kilobases per year, the *y*-axis is age-adjusted DNAmTL and the *x*-axis describes the genotype group of rs4374022. **B** Box plot shows association of *TESPA1* mRNA expression in the hippocampus brain tissue across genotype groups (*p* = 0.04), The effect allele C is associated with decreased mRNA expression. The *y*-axis is a log2 transformed expression scale. **C**, **D** The bar plots show estimated means left and right hippocampus volumes among AUD sample. **C** Decreased left hippocampus volumes is associated with the number of allele C (additive effect: *p* = 0.002), **D** Decreased right hippocampus volumes is associated with the number of allele C (additive effect: *p* = 0.01).

Each Manhattan plot of EA and AA GWAS results is shown separately in Supplementary Figs. S2–3 and Table S6–7. We found no evidence for genomic inflation in each EA (*λ*_*GC*_ = 1.01) and AA studies (*λ*_*GC*_ = 1.04). We then removed SNPs that had substantial heterogeneity between the GWAS results of EA and AA (*I*^2^ > 75, *P* < 0.05). The total number of SNPs for meta GWAS analysis was 7,230,852. Interestingly, at the genome-wide significant level, meta-analysis of GWAS for DNAmTL showed that the intronic SNP, rs4374022 in *TESPA1* was significantly associated with DNAmTL shortening by 0.058 kilobases per year (*p* = 3.9 × 10^−8^) with each additional copy of the major allele C (Figs. 2A, B, 3A, Table 2) and the additional 18 imputed SNPs in high LD with rs4374022 in *TESPA1* were observed at the genome-wide level (Table 2).

**Table 2:** GWAS meta-analysis of DNAmTL derived from participants of European Ancestry and African Ancestry.

SNP	CHR	BP	Effect allele^a^	META			EA		AA		Gene or nearest gene
				BETA (EFFECT)	Meta *P*-value	*P* (I^2^)	MAF (MA)	Association *P*-value	MAF(MA)	Association *P*-value	
Genotyped SNPs (*p* < 1 × 10^−5^)											
rs4374022	12	55,373,780	C	−0.058	3.87E−08	0.81(0)	0.22(T)	0.00014	0.48 (T)	0.00012	*TESPA1*
rs3759165	12	55,367,715	C	−0.054	2.23E−07	0.63 (0)	0.22 (T)	0.00016	0.46 (C)	0.0005	*TESPA1*
rs2147317	1	68,990,012	C	−0.052	1.17E−06	0.65 (0)	0.35 (C)	0.00058	0.25 (C)	0.00072	*DEPDC1-AS1*
rs8128746	21	19,656,171	C	−0.049	1.84E−06	0.61 (0)	0.43 (C)	9.24E-05	0.28 (C)	0.0073	*TMPRSS15*
rs7297842	12	55,404,523	A	−0.050	3.25E−06	0.36 (0)	0.21 (G)	0.00017	0.47 (A)	0.0051	*TESPA1; NEUROD4*
rs842192	1	1.78E + 08	C	−0.055	4.47E−06	0.99 (0)	0.13 (A)	0.0082	0.34 (A)	0.0002	*SEC16B*
rs3776566	5	36,631,733	G	−0.061	5.48E−06	0.75 (0)	0.05 (T)	0.02	0.29 (T)	0.0001	*SLC1A3*
rs6682188	1	68,984,394	T	−0.055	5.79E−06	0.65 (0)	0.18 (T)	0.0026	0.21 (T)	0.0008	*DEPDC1-AS1*
rs17082453	4	54,011,603	T	−0.109	5.84E−06	0.57 (0)	0.07 (T)	0.00072	0.03 (T)	0.0027	*SCFD2*
rs7259473	19	39,493,010	G	−0.045	7.31E−06	0.63 (0)	0.49 (G)	0.0026	0.33 (G)	0.001	*FBXO17;FBXO27*
rs3851604	12	55,381,991	G	−0.049	7.33E−06	0.30 (6.1)	0.21 (A)	0.00017	0.45 (A)	0.01	*TESPA1;NEUROD4*
rs2477083	1	68,921,521	G	−0.055	7.56E−06	0.55 (0)	0.14 (G)	0.00039	0.23 (G)	0.006	*RPE65;DEPDC1*
rs4738003	8	70,542,552	C	−0.045	7.77E−06	0.70 (0)	0.48 (C)	0.00038	0.31 (C)	0.0077	*SULF1*
rs2555454	2	64,522,307	A	−0.054	8.23E−06	0.12 (58.4)	0.31 (A)	0.0055	0.12 (A)	0.00018	*LOC100507006; MIR4433B*
rs7978952	12	55,397,643	T	−0.047	9.44E−06	0.27 (18.9)	0.22 (C)	0.00015	0.39 (T)	0.014	*TESPA1;NEUROD4*
Imputed SNPs (*p* < 5 × 10^−8\^)											
rs1408065	6	133,192,978	A	−0.071	4.49E−08	0.51(0)	0.24(A)	5.56E-07	0.08(A)	0.04293	*RPS12;LINC00326*
rs7973023	12	55,357,264	C	−0.059	1.95E−08	0.59(0)	0.23(T)	3.53E-05	0.47(C)	0.000204	*TESPA1*
rs7973141	12	55,357,382	C	−0.059	2.24E−08	0.65(0)	0.23(T)	5.03E-05	0.47(C)	0.000171	*TESPA1*
rs7973347	12	55,357,387	G	−0.059	2.24E−08	0.65(0)	0.23(C)	5.03E-05	0.47(G)	0.000171	*TESPA1*
rs12301448	12	55,358,691	T	−0.058	2.76E−08	0.60(0)	0.23(C)	4.91E-05	0.46(T)	0.000207	*TESPA1*
rs1532052	12	55,361,314	A	−0.058	2.03E−08	0.70(0)	0.23(G)	6.94E-05	0.46(A)	0.000115	*TESPA1*
rs10783706	12	55,362,002	G	−0.058	2.45E−08	0.81(0)	0.23(A)	0.000118	0.46(G)	8.51E−05	*TESPA1*
rs10876674	12	55,362,156	C	−0.057	4.60E−08	0.75(0)	0.23(T)	0.000118	0.46(C)	0.000153	*TESPA1*
rs10876678	12	55,369,024	A	−0.059	2.07E−08	0.98(0)	0.24(G)	0.000163	0.47(G)	5.43E−05	*TESPA1*
rs1565170	12	55,369,858	C	−0.061	7.29E−09	0.96(0)	0.23(T)	0.000118	0.44(T)	2.72E−05	*TESPA1*
rs1565171	12	55,369,960	C	−0.061	7.29E−09	0.96(0)	0.23(A)	0.000118	0.44(A)	2.72E−05	*TESPA1*
rs1565172	12	55,370,164	C	−0.061	7.29E−09	0.96(0)	0.23(T)	0.000118	0.44(T)	2.72E−05	*TESPA1*
rs7314905	12	55,370,782	G	−0.058	4.71E−08	0.80(0)	0.23(C)	0.000135	0.47(C)	0.000141	*TESPA1*
rs7314940	12	55,370,948	C	−0.059	3.39E−08	0.88(0)	0.23(T)	0.000135	0.42(T)	0.000104	*TESPA1*
rs10876679	12	55,371,263	C	−0.061	8.56E−09	0.96(0)	0.23(A)	0.000135	0.43(A)	2.80E−05	*TESPA1*
rs7315419	12	55,371,307	C	−0.061	8.53E−09	0.96(0)	0.23(G)	0.000135	0.43(G)	2.78E−05	*TESPA1*
rs2046686	12	55,371,671	C	−0.061	8.53E−09	0.96(0)	0.23(T)	0.000135	0.43(T)	2.78E−05	*TESPA1*
rs11171205	12	55,372,239	A	−0.058	4.71E−08	0.80(0)	0.23(G)	0.000135	0.47(G)	0.000141	*TESPA1*
rs1488039	12	55,373,112	G	−0.058	4.71E−08	0.80(0)	0.23(A)	0.000135	0.47(A)	0.000141	*TESPA1*

*EA* European Ancestry participants, *AA* Africa Ancestry participants, *META* GWAS meta-analysis, *CHR* chromosome, *BP* base pair (physical location) on genome (hg19), *MAF* minor allele frequency, *P*-value present refers to association test with EAA. *I*^*2*^ *(P)* heterogeneity index with *P*-value, *TESPA1* Thymocyte Expressed, Positive Selection Associated 1, *SLC1A3* solute carrier family 1 member 3, *DEPDC1-AS1* DEP Domain Containing 1-Antisense RNA 1, *TMPRSS15* Transmembrane Serine Protease 15, *SULF1* Sulfatase 1, *NPHP3* Nephrocystin 3, *ACAD11* Acyl-CoA Dehydrogenase Family Member 11, *SEC16B* SEC16 Homolog B, Endoplasmic Reticulum Export Factor, *SCFD2* Sec1 Family Domain Containing.

^a^Effect Allele corresponds to the effect size’s sign; it may not be the minor allele.

**Fig. 4:**
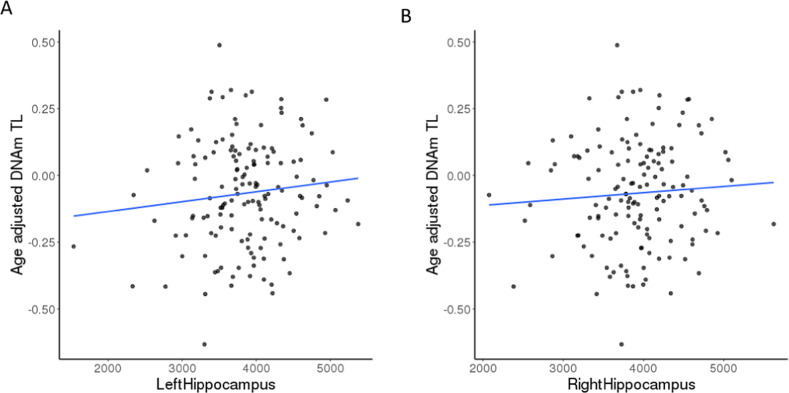
Association of DNAmTL with hippocampus volumes among individuals with AUD. **A** Correlations between age-adjusted DNAmTL and left hippocampus volume among AUD (*n* = 144). The line was a linear fit which age-adjusted DNAmTL was regressed on left hippocampus volume (*β* = 5.6 × 10^−5^, *p* = 0.02) after adjusting for sex, race, blood cell counts, smoking status, BMI, and eTIV. **B** Correlations between age-adjusted DNAmTL and right hippocampus volume. The line was a linear fit which DNAmTL was regressed on right hippocampus volume (*β* = 4.5 × 10^−5^, *p* = 0.07) in the full model with additional adjustment for eTIV.

Cis-eQTL analysis using BRAINEAC expression data showed that the C allele (AF = 78% in EA, 52% in AA) of the genome-wide significant SNP, rs4374022 associated with a higher DNAmTL shortening rate also had a significant association with decreased *TESPA1* mRNA expression in hippocampal tissue from postmortem brains (Fig. 3B, *β* = −0.37, *p* = 0.04). The structural neuroimaging analysis also showed that the C allele was correlated with smaller left and right hippocampus volumes in the AUD sample (*p* < 0.01, Fig. 3C, D). Given that *TESPA1* SNP rs4374022 was associated with DNAm TL shortening and smaller hippocampus volumes and decreased mRNA expression in BRAINEAC data, further analysis revealed that DNAmTL shortening among AUD cases was also related to reduced volumes in hippocampus (Fig. 4A, B, *β* = 5.6 × 10^5^, *p* < 0.05), a key brain region implicated in aging-associated cognitive decline [53, 54].

## Discussion

This is the first study using DNAmTL to investigate the impact of AUD and alcohol-associated clinical phenotypes on TL-related biological aging. Our study revealed that cellular aging as indicated by DNAmTL shortening, is accelerated in the AUD group compared to HC (0.11 kilobases per year shorter in AUD, *p* *=* 4.0 × 10^−12^). Additionally, we found significant associations between DNAmTL shortening and heavy alcohol consumption measured by average drinks per day, the number of heavy drinking days, and total drinks in past 90 days (Table 1, ps < 0.001) suggesting a dose-response relationship. Further clinical analyses substantiated our findings and showed that DNAmTL shortening was significantly associated with elevated GGT and AST, clinical measures that often indicate a more severe AUD phenotype or worse disease progression [8, 55, 8, 56]. These findings are in line with previous studies that show more severe AUD phenotypes are associated with accelerated biological aging [11, 12] and brain aging [57]. Importantly, findings were replicated in a general population showing that alcohol consumption is associated with shortened DNAmTL.

While previous reports have shown an association of shortened TL in AUD [23, 58, 59], we used a novel approach to predict TL via DNA methylation. To confirm the validity of this approach, we compared DNAmTL with conventionally measured TL in a subset of our NIAAA sample and found positive correlations (Fig. S1, *R* = 0.3, *p* = 2.2 × 10^−05^). Lu et al. [28] found that DNAmTL outperformed TL in showing stronger associations with age, sex, ethnicity, lifestyle factors (i.e., smoking, BMI, etc.) and several clinical biomarkers. In addition, DNAmTL had a better predictive power than TL in capturing a correlation between time to death and time to coronary heart disease or heart failure, as well as age-at-menopause and dietary factors [28]. Considering the technical complexities of traditional TL assessments, DNAmTL is more robust and cost-effective for studying age-related health outcomes [28] and captures more biological variation of blood cell counts and is more strongly related to biomarkers of immunosenescence than measured TL [28]. DNAmTL is also an important epigenetic biomarker, which captures additional aspects of molecular aging, in particular the link between cell replication and age-related diseases. Importantly, the 140 CpGs we used to predict DNAmTL did not overlap with any previously identified alcohol-related CpGs (2504 CpGs for alcohol consumption [60], 96 for AUD [29]), reducing the likelihood that alcohol might have a confounding influence on measuring methylation-based TL.

To further elucidate the underlying molecular mechanisms and genetic factors contributing to DNAmTL shortening in AUD [61], we conducted GWAS on DNAmTL. Our GWAS meta-analyses identified a genome-wide significant SNP rs4374022 and several suggestive SNPs in *TESPA1*, which is highly expressed in several brain regions (https://www.proteinatlas.org). TESPA1 is involved in the development and maturation of T-cells and plays a critical role in immune defects in the elderly [62]. Furthermore, animal models have shown that *TESPA1-*knockout mice (*Tespa1*^−/−^, KO) exhibit more severe inflammation [63]. Given that a critical component of aging is a set of functional and structural changes in the immune system that manifests in reduced B and T cell production [62], genetic variation in *TESPA1* might influence the aging process. This hypothesis is intriguing as *TESPA1* has also been identified as one of the top ten differentially expressed genes in a recent study investigating differential gene expression Alzheimer’s disease (AD) (compared to healthy controls) in several brain regions [64]. Furthermore, TESPA1 is involved in the Ca^2+^ transfer from endoplasmic reticulum (ER) to mitochondria (MT) [65]. The molecular interactions between ER and mitochondria membrane, referred as the MT-ER contacts, may play a crucial role in aging and in the development of aging-associated diseases derived from mitochondrial dysfunction as consequences of oxidative stress [66–68]. Genetic variation in *TESPA1* might further influence the effects of heavy alcohol consumptions on immune and mitochondrial function, but additional studies are needed to substantiate this hypothesis.

Biological validation and follow-up analyses using our structural neuroimaging data and cis-eQTL data showed that the genome-wide significant *TESPA1* SNP, rs4374022, was also associated with decreased hippocampal volumes and reduced mRNA expression in hippocampal tissue from postmortem brain. This is the first evidence that *TESPA1* might be associated with brain volume regulation, possibly via regulation of the immune system such as modulating T-cell infiltration in hippocampus [69]. This finding is intriguing as genes regulating the immune system have been implicated in cognitive decline and Alzheimer’s disease [70–72].

In line with our finding that genetic variation in *TESPA1* was associated with decreased mRNA expression in hippocampus, we observed that DNAmTL shortening was associated in general with reduced hippocampal volume among AUD cases (Fig. 4, *p* < 0.05). While several brain regions have been shown to be associated with AUD and aging, the hippocampus is one of the brain regions most affected by aging and the volume loss is estimated to reach up to 35% over the age range of 30–90 years [73]. Alcohol misuse might accelerate hippocampal volume loss and lead to cognitive decline over time [74]. While the exact mechanisms of brain volumes loss due to heavy alcohol exposure are unknown, our findings suggest that aging-related changes in the immune system regulated via *TESPA1* in conjunction with heavy alcohol use might lead to advanced cellular aging, structural brain changes, and subsequent cognitive declines. Future studies might be necessary to investigate these possibilities. The Meta-Analysis Gene-set Enrichment of Variant Association (MAGENTA) analysis [75] was used to compare the results of GWAS meta-analysis with gene sets of pre-specified pathways related to aging and addiction, showing the top 5% findings involved in the pathological mechanism of alcoholism and other addictions and of disorders of dopaminergic and serotonergic pathways (Table S8). Our top 25% findings significantly exhibited more gene enrichments in long-term depression and drug addictions pathways. The findings imply that the suggestive variants may be polygenic and relevant to shared functional biological pathways with the aging process in addiction.

Our study has several strengths, including a well-characterized sample and replication cohort, enabling us to conduct various endophenotypic analyses, including degree of disease severity analyses. In addition to enrichment of clinical phenotypes, structural neuroimaging data added power to validate biological function. Second, all our findings exhibited consistent support in downstream biological analyses. The variant contributing to DNAmTL shortening also showed loss of hippocampus volume and decreased mRNA expression, which could help elucidate the relationship between the neuronal immune system and biological aging. Despite the strengths, our findings do not demonstrate whether AUD is a predisposing factor for shortened DNAmTL or if it is a consequence of chronic heavy alcohol use. To address this limitation of our cross-sectional study, future studies could consider collections of methylation data at the multiple time points to investigate the effect of long-term alcohol use on DNAmTL shortening [76]. Furthermore, our study was limited in collecting data on other environmental factors such as exercise and diet that may influence DNA methylation levels of telomere length and were not included in the analyses, although we accounted for age, sex, race, smoking status, and BMI. Our study had adequate power to detect the association of DNAmTL with alcohol drinking behaviors, but was underpowered for each ancestry-specific GWAS. Our cross-ancestry GWAS meta-analysis identified genome-wide significant variants associated with acceleration in DNAmTL shortening, although we failed to replicate our findings in the GS cohorts, likely due to differences in sample characteristics including phenotypic and genetic heterogeneity [77]. Future studies may be needed to detect additional common and rare functional variants with large effects in a cross-ancestry GWAS setting.

In conclusion, DNAmTL shortening was associated with AUD and heavy alcohol consumption behaviors including alcohol-related clinical endophenotypes. In addition, we identified an association between *TESPA1* and DNAmTL shortening processes, indicating a potential role of the immune system on biological aging. Further important lines of investigation include the analysis of shared mechanisms underlying heavy alcohol use and age-related DNAmTL, which could lead to novel methods for detection and treatment of aging-related morbidities.

## Supplementary information

Supplementary Information
