# Supplementary material for: Alcohol use disorder is associated with DNA methylation-based shortening of telomere length and regulated by TESPA1: implications for aging
Source: Mol Psychiatry. 2022 Jun 15;27(9):3875–84. doi: 10.1038/s41380-022-01624-5 (PMC9708583; doi:10.1038/s41380-022-01624-5)
Supplement: Supplementary file 1 — Supplementary Information [file 41380_2022_1624_MOESM1_ESM.docx]

**Supplementary Information for**

**Alcohol Use Disorder is associated with DNA methylation-based shortening of telomere length and regulated by *TESPA1*: implications for aging**

Jeesun Jung, Ph.D.^1^, Daniel L. McCartney, Ph.D.^2^, Josephin Wagner, M.D.^1^, Daniel B. Rosoff^1^, Melanie Schwandt, Ph.D.^3^, Hui Sun, M.D.^3^, Corinde E. Wiers, Ph.D.^4^, Luana Martins de Carvalho, Ph.D.^4^, Nora D. Volkow, M.D.^4^, Rosie M. Walker, Ph.D. ^2^, Archie Campbell, M.A.^2^, David J. Porteous, Ph.D. ^2^, Andrew M. McIntosh, M.D.^2^ Riccardo E. Marioni, Ph.D.^2^ Steve Horvath, Ph.D.^5^, Kathryn L. Evans, Ph.D. ^2^, Falk W. Lohoff, M.D.^1*^

^1^Section on Clinical Genomics and Experimental Therapeutics, National Institute on Alcohol Abuse and Alcoholism, National Institutes of Health, Bethesda, MD, USA

^2^Centre for Genomic and Experimental Medicine, Institute of Genetics and Cancer, University of Edinburgh, Edinburgh, United Kingdom

^3^Office of the Clinical Director, National Institute on Alcohol Abuse and Alcoholism, National Institutes of Health, Bethesda, MD, USA

^4^ Laboratory of Neuroimaging, National Institute on Alcohol Abuse and Alcoholism, National Institutes of Health, Bethesda, MD, USA

^5^Department of Biostatistics, Fielding School of Public Health, University of California Los Angeles, Los Angeles, CA, USA

^6^Department of Human Genetics, David Geffen School of Medicine, University of California Los Angeles, Los Angeles, CA, USA

^*^ Corresponding Author:

Falk W. Lohoff, M.D.

Chief, Section on Clinical Genomics and Experimental Therapeutics (CGET)

Lasker Clinical Research Scholar

National Institute on Alcohol Abuse and Alcoholism (NIAAA)

National Institutes of Health

10 Center Drive (10CRC/2-2352)

Bethesda, MD 20892-1540

Office: 301-827-1542

falk.lohoff@nih.gov

**Supplementary Figures and Tables**

- Supplementary methods: Replication Study: Generation Scotland Sample (Scottish Family Health Study)
- Supplementary Tables

Table S1: Sample characteristics

Table S2: Association of DNAmTL with alcohol use and liver function enzyme biomarkers among individuals with AUD

Table S3: Association of DNAmTL with alcohol use and liver function enzyme biomarkers without any substance use disorders (SUD) (Sensitivity Analysis)

Table S4: Sex-specific association of DNAmTL with alcohol use and liver function enzyme biomarkers

Table S5: Association of DNAmTL with alcohol use and liver function enzyme biomarkers after controlling for comorbid disorders

Table S6: GWAS of DNAm TL in EA (listed by SNPs <1.0E-06 that have suggestive association)

Table S7: GWAS of DNAm TL in AA (listed by SNPs <1.0E-06 that have suggestive association)

Table S8: Gene set enrichment analysis by MAGENTA with genes above 95% percentile of the p-value

- Supplementary Figures

Figure S1: Correlation of Leukocyte Telomere Length (T/S) with DNAm Telomere Length

Figure S2: Manhattan plot for GWAS with European Ancestry (EA) individuals

Figure S3: Manhattan plot for GWAS with African Ancestry (AA) individuals

**Supplementary methods of replication study**

**Generation Scotland: Scottish Family Health Study (GS, Set 1: n=2,578, Set 2: n=4,450)**

*Cohort description:* Generation Scotland is a family-structured, population-based cohort study of over 24,000 people from across Scotland, aged between 18 and 99 years at the study baseline (2006-2011). A broad set of phenotype data were collected at baseline and data linkage to electronic health records has enabled follow-up to collect information on incident disease outcomes. Full details have been reported previously ^4, 5^. DNA was obtained from whole blood at the study baseline. For set 1, a genetic relationship matrix was built using GCTA-GRM, and a relatedness coefficient of <0.025 was specified to exclude related individuals and we used only unrelated subject (n=1501). Set 2 are unrelated (genetic relatedness <0.05) to each other, and to the participants from Set 1 (n=2718). The quality control steps for Set 1 and Set 2 were nearly identical to one another and full details have been reported previously.

*DNA Methylation*: DNA methylation from whole blood was assessed using the Illumina EPIC array. To date, the arrays have been run on two tranches of the GS data: Sample size of set 1 is 2,578 and that of set 2 was 4,450. Full details on quality control have been reported previously ^6, 7^. Briefly, probes were filtered based on three criteria: outliers based on visual inspection of the log median intensity of the methylated versus unmethylated signal per array; a beadcount <3 in more than 5% of samples; and more than 5% of samples have a detection p-value >0.05. Samples were removed if predicted sex did not match reported sex and if >1% of CpGs had a detection p-value >0.05. These data were then uploaded to Horvath’s online calculator, as described in the main text, to generate the various clock estimates. All components of Generation Scotland received ethical approval from the NHS Tayside Committee on Medical Research Ethics (REC Reference Number: 05/S1401/89). All participants provided broad and enduring written informed consent for biomedical research. Generation Scotland has also been granted Research Tissue Bank status by the East of Scotland Research Ethics Service (REC Reference Number: 20/ES/0021), providing generic ethical approval for a wide range of uses within medical research. This study was performed in accordance with the Helsinki declaration.

**Supplementary Table S1. Sociodemographic characteristics of study participants**

|  | **HC (n=243)** | | **AUD (n=372)** | | **p-value** |
| --- | --- | --- | --- | --- | --- |
|  | N | % | N | % |  |
| Gender |  |  |  |  | 0.005 |
| Male | 118 | 48.56 | 224 | 60.22 |  |
| Race |  |  |  |  | 0.14 |
| White | 133 | 54.73 | 191 | 51.34 |  |
| Black | 103 | 42.39 | 177 | 47.58 |  |
| Others | 7 | 2.88 | 4 | 1.08 |  |
| Smoking | 13 | 5.35 | 233 | 62.63 | <0.0001 |
| Opioid Dependence | 0 | 0 | 30 | 8.5 | <0.0001 |
| Cocaine Dependence | 0 | 0 | 91 | 25.8 | <0.0001 |
| Cannabis Dependence | 4 | 1.75 | 70 | 19.3 | <0.0001 |
| Hallucinogen Dependence | 0 | 0 | 12 | 3.4 | 0.005 |
| Amphetamine Dependence | 0 | 0 | 13 | 3.7 | 0.002 |
| Major Depression | 19 | 8.33 | 105 | 29.8 | <0.0001 |
| Any anxiety disorder | 1 | 0.44 | 59 | 15.9 | <0.0001 |
| Bipolar Disorder | 1 | 0.44 | 9 | 2.6 | 0.1 |
|  | Mean | SD | Mean | SD |  |
| Age | 35.41 | 12.52 | 44.52 | 10.99 | <0.0001 |
| BMI | 26.31 | 4.74 | 27.45 | 5.81 | 0.008 |
| Total Drinks | 51.33 | 113.1 | 937.52 | 700.0 | <0.0001 |
| No of Drink Days | 18.58 | 21.36 | 72.95 | 21.19 | <0.0001 |
| Avg Drinks Per Day | 2.16 | 1.57 | 12.62 | 8.01 | <0.0001 |
| Heavy Drink Days | 3.51 | 11.88 | 64.08 | 27.41 | <0.0001 |
| ADS Score | 0.84 | 1.76 | 20.03 | 8.45 | <0.0001 |
| GGT | 26.05 | 27.66 | 141.50 | 235.23 | <0.0001 |
| ALT | 23.54 | 19.18 | 50.32 | 54.05 | <0.0001 |
| AST | 21.39 | 15.74 | 53.70 | 64.14 | <0.0001 |

Note: AUD = alcohol use disorder; GGT = gamma-glutamyl transferase; ALT = alanine aminotransferase; AST = aspartate aminotransferase; Heavy drinking days are defined as ≥4 drinks a day for females; ≥5 drinks a day for males. ADS=Alcohol Dependence Scale

**Table S2. Association of DNAmTL with alcohol use and liver function enzyme biomarkers among individuals with AUD**

|  | | | | | | | |
| --- | --- | --- | --- | --- | --- | --- | --- |
|  | Basic Model | | |  | Full Model | | |
| **Predictor** | *β* | *SE* | ***P*-value** |  | *β* | *SE* | ***P*-value** |
| Total Drinks | -0.043 | 0.009 | 7.35E-07 |  | -0.033 | 0.008 | 8.32E-05 |
| No of Drinking Days | -0.032 | 0.014 | 0.02 |  | -0.019 | 0.013 | 0.15 |
| Avg Drinks Per Day | -0.038 | 0.009 | 2.02E-05 |  | -0.030 | 0.009 | 0.0006 |
| Heavy Drinking Days | -0.035 | 0.012 | 0.003 |  | -0.020 | 0.012 | 0.08 |
| ADS Score | -0.003 | 0.001 | 0.03 |  | -0.002 | 0.001 | 0.07 |
| GGT | -0.019 | 0.007 | 0.01 |  | -0.020 | 0.007 | 0.005 |
| ALT | -0.007 | 0.008 | 0.38 |  | -0.006 | 0.007 | 0.42 |
| AST | -0.018 | 0.008 | 0.02 |  | -0.017 | 0.007 | 0.015 |
| *Note*: GGT = gamma-glutamyl transferase; ALT = alanine aminotransferase; AST = aspartate aminotransferase; ADS=Alcohol Dependence Scale; Heavy drinking days are defined as ≥4 drinks a day for females; ≥5 drinks a day for males. Adjusted for age, gender, race, six blood cell counts in the basic model, and additionally adjusted for smoking status, and body mass index in the full model. | | | | | | | |

**Table S3: Association of DNAmTL with alcohol use and liver function enzyme biomarkers without any substance use disorders (SUD)** **(Sensitivity Analysis)**

|  | | Basic Model | | | | | Full Model | | |
| --- | --- | --- | --- | --- | --- | --- | --- | --- | --- |
|  | *β* | | *SE* | ***P*-value** |  | *β* | | *SE* | ***P*-value** |
| **NIAAA AUD without any SUD (n=212)** | | | | | | | | | |
| Total Drinks | -0.03 | | 0.012 | **0.01** |  | -0.022 | | 0.011 | 0.06 |
| No of Drinking Days | -0.013 | | 0.012 | 0.28 |  | -0.005 | | 0.011 | 0.63 |
| Average Drinks Per Day | -0.022 | | 0.012 | 0.06 |  | -0.016 | | 0.011 | 0.15 |
| Heavy Drinking Days | -0.016 | | 0.012 | 0.17 |  | -0.006 | | 0.011 | 0.59 |
| ADS Score | -0.03 | | 0.014 | **0.04** |  | -0.025 | | 0.013 | 0.06 |
| GGT | -0.01 | | 0.01 | 0.39 |  | -0.017 | | 0.011 | 0.14 |
| ALT | -0.01 | | 0.01 | 0.37 |  | -0.012 | | 0.012 | 0.31 |
| AST | -0.02 | | 0.01 | 0.08 |  | -0.023 | | 0.01 | **0.04** |
| **NIAAA AUD with SUD (n=141)** | | | | | | | | | |
| Total Drinks | -0.06 | | 0.013 | **6.5E-05** |  | -0.05 | | 0.014 | **0.0004** |
| No of Drinking Days | -0.03 | | 0.014 | **0.04** |  | -0.02 | | 0.014 | 0.13 |
| Average Drinks Per Day | -0.05 | | 0.014 | **0.0002** |  | -0.05 | | 0.014 | **0.0008** |
| Heavy Drinking Days | -0.04 | | 0.014 | **0.005** |  | -0.03 | | 0.014 | **0.03** |
| ADS Score | -0.01 | | 0.016 | 0.40 |  | -0.015 | | 0.016 | 0.34 |
| GGT | -0.04 | | 0.014 | **0.005** |  | -0.04 | | 0.013 | **0.007** |
| ALT | 0.004 | | 0.015 | 0.80 |  | 0.004 | | 0.014 | 0.77 |
| AST | -0.02 | | 0.014 | 0.22 |  | -0.013 | | 0.014 | 0.34 |

*Note*: AUD = alcohol use disorder; GGT = gamma-glutamyl transferase; ALT = alanine aminotransferase; AST = aspartate aminotransferase; ADS=Alcohol Dependence Scale; Heavy drinking days are defined as ≥4 drinks a day for females; ≥5 drinks a day for males. Adjusted for age, sex, race, AUD diagnosis, five blood cell counts in the basic model, and additionally adjusted for smoking status, and body mass index in the full model.

**Table S4: Sex-specific association of DNAmTL with alcohol use and liver function enzyme biomarkers**

|  |  | **Males (n=342)** | | | |  | **Females (n=273)** | | | |
| --- | --- | --- | --- | --- | --- | --- | --- | --- | --- | --- |
|  |  | *β* | *SE* | ***P*-value** | |  | *β* | | *SE* | ***P*-value** |
| **NIAAA all sample^*^** | | | | | | | | | | |
| Total Drinks |  | -0.042 | 0.01 | | **6.4E-05** |  | -0.06 | 0.01 | | **1.1E-05** |
| No of Drinking Days |  | -0.007 | 0.01 | | 0.60 |  | -0.051 | 0.016 | | **0.002** |
| Average Drinks Per Day |  | -0.040 | 0.011 | | **0.0002** |  | -0.046 | 0.014 | | **0.002** |
| Heavy Drinking Days |  | -0.027 | 0.013 | | **0.036** |  | -0.067 | 0.018 | | **0.0003** |
| ADS Score |  | -0.041 | 0.016 | | **0.009** |  | -0.043 | 0.023 | | 0.06 |
| GGT |  | -0.035 | 0.009 | | **0.0001** |  | -0.015 | 0.01 | | 0.15 |
| ALT |  | -0.014 | 0.009 | | 0.13 |  | -0.015 | 0.011 | | 0.16 |
| AST |  | -0.028 | 0.009 | | **0.002** |  | -0.022 | 0.01 | | **0.04** |
| **AUD only** | | | | | | | | | | |
|  |  | **Males (n=224)** | | | |  | **Females (n=148)** | | | |
| Total Drinks |  | -0.04 | 0.01 | | **0.0002** |  | -0.06 | 0.014 | | **5.6E-05** |
| No of Drinking Days |  | -0.015 | 0.01 | | 0.17 |  | -0.036 | 0.015 | | **0.02** |
| Average Drinks Per Day |  | -0.036 | 0.011 | | **0.001** |  | -0.046 | 0.015 | | **0.003** |
| Heavy Drinking Days |  | -0.02 | 0.011 | | 0.09 |  | -0.045 | 0.015 | | **0.003** |
| ADS Score |  | -0.03 | 0.012 | | **0.04** |  | -0.026 | 0.017 | | 0.13 |
| GGT |  | -0.04 | 0.011 | | **0.0005** |  | -0.016 | 0.015 | | 0.31 |
| ALT |  | -0.008 | 0.012 | | 0.52 |  | -0.018 | 0.015 | | 0.24 |
| AST |  | -0.028 | 0.012 | | **0.015** |  | -0.026 | 0.015 | | 0.08 |
| ^*^Note: p-value was calculated based on the basic model with additional adjustment for AUD | | | | | | | | | | |

| **Table S5: Association of DNAmTL with alcohol use and liver function enzyme biomarkers after controlling for comorbid disorders** | | | | | | | | | | | | | | | |
| --- | --- | --- | --- | --- | --- | --- | --- | --- | --- | --- | --- | --- | --- | --- | --- |
|  | Any Drug Dependence^*2^ | | | | |  | Any Mood Disorder^*3^ | | | |  | Any Psychiatric Disorder^*4^ | | |  |
|  | | | *β* | *SE* | ***P*-value** |  | *β* | *SE* | | ***P*-value** |  | *β* | *SE* | ***P*-value** |  |
| **NIAAA all sample (n=615)^*1^** | | | | | |  |  | |  |  |  |  |  |  |  |
| Total Drinks | | -0.042 | | 0.009 | **1.1E-06** |  | -0.044 | | 0.008 | **2.9E-07** |  | -0.043 | 0.008 | **4.6E-07** |  |
| No of Drinking Days | | -0.025 | | 0.01 | **0.02** |  | -0.026 | | 0.01 | **0.01** |  | -0.025 | 0.01 | **0.016** |  |
| Average Drinks Per Day | | -0.038 | | 0.009 | **1.7E-05** |  | -0.041 | | 0.009 | **3.8E-06** |  | -0.040 | 0.009 | **7.1E-06** |  |
| Heavy Drinking Days | | -0.04 | | 0.011 | **0.0003** |  | -0.04 | | 0.011 | **0.0001** |  | -0.041 | 0.011 | **0.0002** |  |
| ADS Score | | -0.039 | | 0.013 | **0.004** |  | -0.041 | | 0.014 | **0.003** |  | -0.04 | 0.014 | **0.004** |  |
| GGT | | -0.025 | | 0.007 | **0.0008** |  | -0.026 | | 0.008 | **0.0006** |  | -0.026 | 0.008 | **0.0008** |  |
| ALT | | -0.01 | | 0.007 | 0.18 |  | -0.01 | | 0.007 | 0.15 |  | -0.01 | 0.007 | 0.18 |  |
| AST | | -0.022 | | 0.007 | **0.0026** |  | -0.022 | | 0.007 | **0.002** |  | -0.02 | 0.007 | **0.002** |  |
| **NIAAA AUD sample (n=372)** | | | | | | | | | | | | | | |  |
| Total Drinks | | -0.042 | | 0.009 | **3.4E-06** |  | -0.044 | | 0.009 | **8.4E-07** |  | -0.043 | 0.009 | **2.3E-06** |  |
| No of Drinking Days | | -0.20 | | 0.009 | **0.02** |  | -0.021 | | 0.009 | **0.018** |  | -0.02 | 0.009 | **0.02** |  |
| Average Drinks Per Day | | -0.035 | | 0.009 | **0.0001** |  | -0.038 | | 0.009 | **3.7E-05** |  | -0.036 | 0.009 | **9.4E-05** |  |
| Heavy Drinking Days | | -0.025 | | 0.009 | **0.005** |  | -0.027 | | 0.009 | **0.003** |  | -0.026 | 0.009 | **0.004** |  |
| ADS Score | | -0.022 | | 0.01 | **0.03** |  | -0.024 | | 0.01 | **0.026** |  | -0.022 | 0.01 | **0.04** |  |
| GGT | | -0.027 | | 0.01 | **0.007** |  | -0.028 | | 0.01 | **0.005** |  | -0.027 | 0.01 | **0.006** |  |
| ALT | | -0.007 | | 0.009 | 0.44 |  | -0.008 | | 0.01 | 0.42 |  | -0.007 | 0.01 | 0.46 |  |
| AST | | -0.022 | | 0.009 | **0.02** |  | -0.023 | | 0.009 | **0.016** |  | -0.022 | 0.01 | **0.02** |  |

*Note*: AUD = alcohol use disorder; GGT = gamma-glutamyl transferase; ALT = alanine aminotransferase; AST = aspartate aminotransferase; ADS=Alcohol Dependence Scale; Heavy drinking days are defined as ≥4 drinks a day for females; ≥5 drinks a day for males.

*^1^Adjusted for age, sex, race, AUD diagnosis, five blood cell counts in the basic model.

*2 additionally adjusted for any lifetime Drug Dependence defined any of illicit drug dependence among Opioid, Cocaine, Cannabis, Hallucinogen, Amphetamine dependence in the basic model.

*3 additionally adjusted for lifetime Mood Disorder defined any of major depression disorder, Bipolar disorder, and any anxiety (including generalized anxiety disorder)

*4 additionally adjusted for any psychiatric disorder defined by any lifetime drug dependence + any Mood Disorder

**Supplementary Table S6: GWAS of DNAmTL in EA (listed by SNPs <1.0E-06 that have suggestive association)**

| SNP | CHR | BP | MA | BETA | SE | P-value | MAF | HWE  P-value | Gene Symbol | Function |
| --- | --- | --- | --- | --- | --- | --- | --- | --- | --- | --- |
| rs11088231 | 21 | 16325570 | A | -0.115 | 0.020 | 4.34E-08 | 0.11 | 0.69 | *LINC02246;NRIP1* | intergenic |
| rs525153 | 6 | 133219461 | T | -0.080 | 0.015 | 1.43E-07 | 0.24 | 0.41 | *RPS12;LINC00326* | intergenic |
| rs190020402 | 4 | 135005656 | A | -0.350 | 0.066 | 1.97E-07 | 0.01 | 1.00 | *PCDH10;PABPC4L* | intergenic |
| rs191238079 | 4 | 135022327 | G | -0.350 | 0.066 | 1.97E-07 | 0.01 | 1.00 | *PCDH10;PABPC4L* | intergenic |
| rs141588883 | 4 | 135076759 | A | -0.350 | 0.066 | 1.97E-07 | 0.01 | 1.00 | *PCDH10;PABPC4L* | intergenic |
| rs74963982 | 4 | 135079231 | A | -0.350 | 0.066 | 1.97E-07 | 0.01 | 1.00 | *PCDH10;PABPC4L* | intergenic |
| rs142734744 | 4 | 135148312 | C | -0.350 | 0.066 | 1.97E-07 | 0.01 | 1.00 | *PABPC4L;LINC02462* | intergenic |
| rs184139071 | 4 | 135221113 | A | -0.350 | 0.066 | 1.97E-07 | 0.01 | 1.00 | *PABPC4L;LINC02462* | intergenic |
| rs271110 | 6 | 133232469 | A | -0.078 | 0.015 | 2.10E-07 | 0.27 | 0.85 | *RPS12;LINC00326* | intergenic |
| rs383235 | 6 | 133221575 | C | -0.078 | 0.015 | 2.46E-07 | 0.25 | 0.41 | *RPS12;LINC00326* | intergenic |
| rs869113926 | 6 | 133221969 | G | -0.078 | 0.015 | 2.46E-07 | 0.25 | 0.41 | *RPS12;LINC00326* | intergenic |
| rs374766 | 6 | 133227669 | A | -0.078 | 0.015 | 2.46E-07 | 0.25 | 0.41 | *RPS12;LINC00326* | intergenic |
| rs583847 | 6 | 133219607 | G | -0.078 | 0.015 | 2.66E-07 | 0.25 | 0.54 | *RPS12;LINC00326* | intergenic |
| rs530078646 | 4 | 134655087 | G | -0.319 | 0.061 | 3.48E-07 | 0.01 | 1.00 | *PCDH10;PABPC4L* | intergenic |
| rs9402467 | 6 | 133195740 | G | -0.076 | 0.015 | 3.68E-07 | 0.25 | 0.42 | *RPS12;LINC00326* | intergenic |
| rs271111 | 6 | 133232646 | T | -0.076 | 0.015 | 3.83E-07 | 0.27 | 0.85 | *RPS12;LINC00326* | intergenic |
| rs2264360 | 12 | 55340399 | A | 0.075 | 0.014 | 3.95E-07 | 0.31 | 0.86 | *MUCL1;TESPA1* | intergenic |
| rs397139 | 6 | 133221923 | T | -0.076 | 0.015 | 4.51E-07 | 0.25 | 0.54 | *RPS12;LINC00326* | intergenic |
| rs412154 | 6 | 133224249 | A | -0.076 | 0.015 | 4.51E-07 | 0.25 | 0.54 | *RPS12;LINC00326* | intergenic |
| rs413864 | 6 | 133225039 | A | -0.076 | 0.015 | 4.51E-07 | 0.25 | 0.54 | *RPS12;LINC00326* | intergenic |
| rs271112 | 6 | 133233127 | G | -0.076 | 0.015 | 4.86E-07 | 0.27 | 0.85 | *RPS12;LINC00326* | intergenic |
| rs271179 | 6 | 133210231 | A | -0.075 | 0.015 | 5.08E-07 | 0.25 | 0.42 | *RPS12;LINC00326* | intergenic |
| rs166600 | 6 | 133212008 | T | -0.075 | 0.015 | 5.08E-07 | 0.25 | 0.42 | *RPS12;LINC00326* | intergenic |
| rs9389039 | 6 | 133212974 | A | -0.075 | 0.015 | 5.08E-07 | 0.25 | 0.42 | *RPS12;LINC00326* | intergenic |
| rs528694 | 6 | 133213203 | A | -0.075 | 0.015 | 5.08E-07 | 0.25 | 0.42 | *RPS12;LINC00326* | intergenic |
| rs1408065 | 6 | 133192978 | A | -0.075 | 0.015 | 5.56E-07 | 0.24 | 0.42 | *RPS12;LINC00326* | intergenic |
| rs1380259 | 6 | 133201588 | C | -0.075 | 0.015 | 5.56E-07 | 0.24 | 0.42 | *RPS12;LINC00326* | intergenic |
| rs9493476 | 6 | 133202519 | A | -0.075 | 0.015 | 5.56E-07 | 0.24 | 0.42 | *RPS12;LINC00326* | intergenic |
| rs9321384 | 6 | 133203888 | A | -0.075 | 0.015 | 5.56E-07 | 0.24 | 0.42 | *RPS12;LINC00326* | intergenic |
| rs718508 | 6 | 133204398 | T | -0.075 | 0.015 | 5.56E-07 | 0.24 | 0.42 | *RPS12;LINC00326* | intergenic |
| rs7741839 | 6 | 133204782 | C | -0.075 | 0.015 | 5.56E-07 | 0.24 | 0.42 | *RPS12;LINC00326* | intergenic |
| rs9493478 | 6 | 133205208 | T | -0.075 | 0.015 | 5.56E-07 | 0.24 | 0.42 | *RPS12;LINC00326* | intergenic |
| rs13362640 | 6 | 133206547 | C | -0.075 | 0.015 | 5.56E-07 | 0.24 | 0.42 | *RPS12;LINC00326* | intergenic |
| rs9375928 | 6 | 133207105 | A | -0.075 | 0.015 | 5.56E-07 | 0.24 | 0.42 | *RPS12;LINC00326* | intergenic |
| rs12139876 | 1 | 211742552 | C | -0.336 | 0.066 | 6.52E-07 | 0.01 | 1.00 | *RD3;SLC30A1* | intergenic |
| rs2205241 | 21 | 16319919 | C | -0.098 | 0.019 | 6.99E-07 | 0.14 | 1.00 | *LINC02246;NRIP1* | intergenic |
| rs79594032 | 14 | 20724036 | T | -0.105 | 0.021 | 8.65E-07 | 0.11 | 0.46 | *OR11H4;TTC5* | intergenic |
| rs41485046 | 21 | 16322487 | T | -0.097 | 0.019 | 9.26E-07 | 0.14 | 0.74 | *LINC02246;NRIP1* | intergenic |
| rs10482862 | 21 | 16323835 | C | -0.097 | 0.019 | 9.26E-07 | 0.14 | 0.74 | *LINC02246;NRIP1* | intergenic |

*Note*: EA = European Ancestry participants; DNAmTL = DNA methylation Telomere length; CHR = chromosome; BP = base pair (physical location) on genome; MAF = minor allele frequency; P-value presents for association test with DNAmTL.

**Supplementary Table S7: GWAS of DNAmTL in AA (listed by SNPs < 1.0E-06 that have suggestive association)**

| SNP | CHR | BP | MA | BETA | SE | P-value | MAF | HWE  P-value | Gene Symbol | Function |
| --- | --- | --- | --- | --- | --- | --- | --- | --- | --- | --- |
| rs343957 | 2 | 44942384 | C | -0.119 | 0.0220 | 1.43E-07 | 0.10 | 0.27 | *CAMKMT* | intronic |
| rs6727707 | 2 | 34485558 | A | -0.080 | 0.0151 | 2.60E-07 | 0.31 | 0.24 | *LINC01317* | ncRNAintronic |
| rs570436 | 2 | 45142673 | C | -0.096 | 0.0183 | 3.37E-07 | 0.16 | 0.42 | *CAMKMT;LINC01833* | intergenic |
| rs4683867 | 3 | 100909312 | C | 0.100 | 0.0191 | 3.43E-07 | 0.16 | 0.79 | *ABI3BP;IMPG2* | intergenic |
| rs340496 | 2 | 45134871 | G | -0.124 | 0.0239 | 4.23E-07 | 0.08 | 0.17 | *CAMKMT;LINC01833* | intergenic |
| rs340497 | 2 | 45134900 | G | -0.124 | 0.0239 | 4.23E-07 | 0.08 | 0.17 | *CAMKMT;LINC01833* | intergenic |
| rs2371920 | 2 | 34487168 | A | -0.078 | 0.0151 | 4.39E-07 | 0.31 | 0.31 | *LINC01317* | ncRNA_intronic |
| rs503272 | 20 | 58979510 | A | 0.103 | 0.0201 | 4.96E-07 | 0.12 | 0.31 | *MIR646HG;LOC101928048* | intergenic |
| rs567047 | 2 | 45139093 | A | -0.105 | 0.0204 | 5.30E-07 | 0.11 | 0.27 | *CAMKMT;LINC01833* | intergenic |
| rs13410806 | 2 | 34488716 | A | -0.077 | 0.0152 | 6.08E-07 | 0.31 | 0.18 | *LINC01317* | ncRNA_intronic |
| rs61141809 | 19 | 48584250 | C | -0.077 | 0.0150 | 6.44E-07 | 0.29 | 1.00 | *PLA2G4C* | intronic |
| rs343955 | 2 | 44944877 | G | -0.113 | 0.0224 | 7.40E-07 | 0.09 | 0.26 | *CAMKMT* | intronic |
| rs62161743 | 2 | 34493660 | T | -0.077 | 0.0153 | 7.80E-07 | 0.31 | 0.17 | *LINC01317* | ncRNA_intronic |
| rs11896377 | 2 | 34494144 | T | -0.077 | 0.0153 | 7.80E-07 | 0.31 | 0.17 | *LINC01317* | ncRNA_intronic |
| rs71719794 | 6 | 104710622 | T | -0.081 | 0.0162 | 9.15E-07 | 0.23 | 0.84 | *NONE;HACE1* | intergenic |
| rs4683965 | 3 | 100906501 | G | 0.099 | 0.0198 | 9.23E-07 | 0.15 | 0.79 | *ABI3BP;IMPG2* | intergenic |
| rs9577365 | 13 | 113096019 | T | -0.173 | 0.0345 | 9.80E-07 | 0.04 | 1.00 | *SPACA7;TUBGCP3* | intergenic |
| rs7984604 | 13 | 113097084 | G | -0.173 | 0.0345 | 9.80E-07 | 0.04 | 1.00 | *SPACA7;TUBGCP3* | intergenic |

*Note*: AA =African Ancestry, DNAmTL = DNA methylation Telomere length; CHR = chromosome; BP = base pair (physical location) on genome; MAF = minor allele frequency; P-value presents for association test with DNAmTL.

**Supplementary Table S8: The results of gene set enrichment analysis by MAGENTA with genes above 95% and 75% percentile of the p-value**

| GS | P value (95% cut off) | FDR_P_value (95% cut off) | EXP_#_GENES | OBS_#_GENES | P value (75% cut off) | FDR_P_value (75% cut off) | EXP_#_GENES | OBS_#_GENES |
| --- | --- | --- | --- | --- | --- | --- | --- | --- |
| Alcoholism | 7.70E-03 | 1.11E-01 | 6 | 13 | 8.10E-03 | 3.17E-02 | 30 | 42 |
| Longevity | 6.57E-01 | 6.28E-01 | 4 | 4 | 3.51E-01 | 4.07E-01 | 22 | 24 |
| Mineral absorption | 5.79E-01 | 5.72E-01 | 2 | 2 | 5.30E-05 | 6.00E-04 | 10 | 21 |
| Porphyrin and chlorophyll metabolism | 1.68E-01 | 3.10E-01 | 1 | 3 | 2.54E-01 | 3.02E-01 | 7 | 9 |
| ABC transporters | 8.78E-01 | 7.67E-01 | 2 | 1 | 4.55E-01 | 4.88E-01 | 10 | 11 |
| Renin-angiotensin system | 2.38E-01 | 3.38E-01 | 1 | 2 | 7.69E-02 | 1.01E-01 | 5 | 8 |
| Bile secretion | 4.45E-01 | 5.56E-01 | 3 | 4 | 7.60E-01 | 7.50E-01 | 17 | 15 |
| cAMP signaling pathway | 4.72E-02 | 1.60E-01 | 10 | 16 | 2.71E-02 | 6.21E-02 | 51 | 63 |
| MAPK signaling pathway | 2.79E-01 | 4.00E-01 | 14 | 16 | 5.64E-01 | 6.33E-01 | 68 | 67 |
| Endocytosis | 6.22E-01 | 6.72E-01 | 12 | 11 | 6.98E-01 | 7.47E-01 | 58 | 55 |
| Serotonergic synapse | 2.59E-02 | 1.23E-01 | 5 | 10 | 1.66E-02 | 4.92E-02 | 25 | 35 |
| GABAergic synapse | 2.16E-01 | 2.94E-01 | 4 | 6 | 9.02E-02 | 1.22E-01 | 20 | 26 |
| Dopaminergic synapse | 7.00E-03 | 5.78E-02 | 6 | 13 | 7.52E-02 | 1.10E-01 | 31 | 38 |
| Long-term depression | 5.30E-01 | 5.85E-01 | 3 | 3 | 6.40E-03 | 2.30E-02 | 14 | 23 |
| Longevity regulating pathway | 6.55E-01 | 6.53E-01 | 4 | 4 | 3.46E-01 | 4.30E-01 | 22 | 24 |
| Cocaine addiction | 2.09E-01 | 3.15E-01 | 2 | 4 | 2.81E-02 | 6.64E-02 | 12 | 18 |
| Amphetamine addiction | 3.26E-02 | 9.79E-02 | 3 | 7 | 1.30E-03 | 9.70E-03 | 16 | 27 |
| Morphine addiction | 1.46E-01 | 2.97E-01 | 4 | 7 | 4.59E-02 | 7.83E-02 | 22 | 29 |
| Nicotine addiction | 2.09E-01 | 2.96E-01 | 2 | 4 | 3.17E-02 | 6.97E-02 | 12 | 18 |
| Cellular senescence | 7.67E-01 | 7.29E-01 | 8 | 6 | 8.36E-01 | 8.04E-01 | 38 | 33 |

**Supplementary Figure S1: Correlation of Leukocyte Telomere Length (T/S) with DNAm Telomere Length**

**
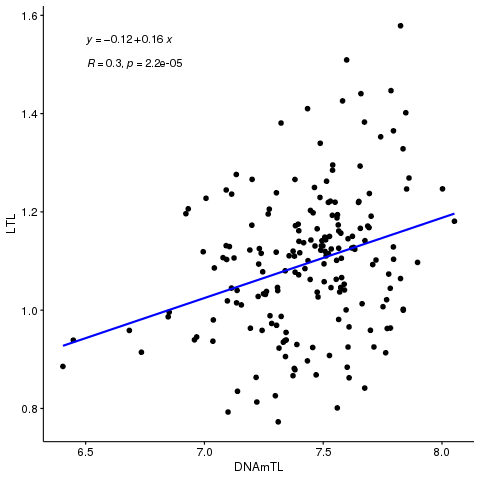
**

**Supplementary Figure S2: Manhattan plot for GWAS with European Ancestry (EA) individuals**

**
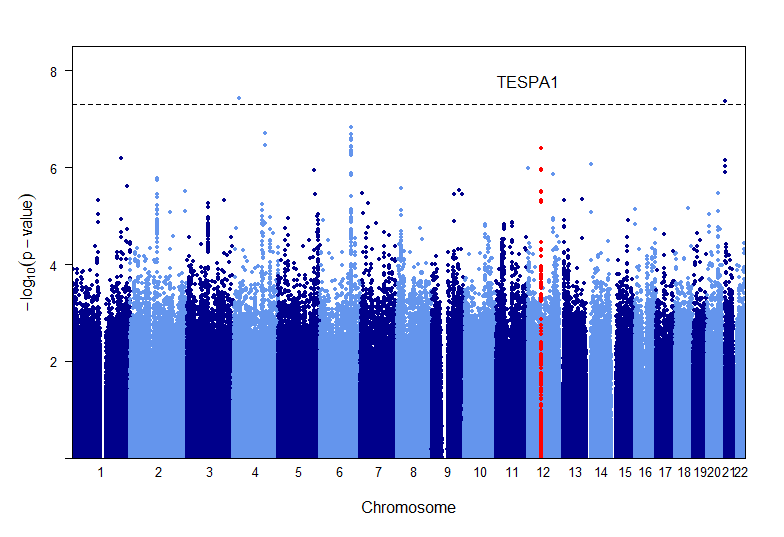
**

The plot shows P-values of all SNPs tested for association with epigenetic age acceleration. The y-axis presents log base 10 transformed P-value. The horizontal dashed line corresponds to the genome-wide association threshold (p=5 x 10^-8^). All SNPs in *TESPA1* were colored with red.

**Supplementary Figure S3: Manhattan plot for GWAS with African Ancestry (AA) individuals**

**
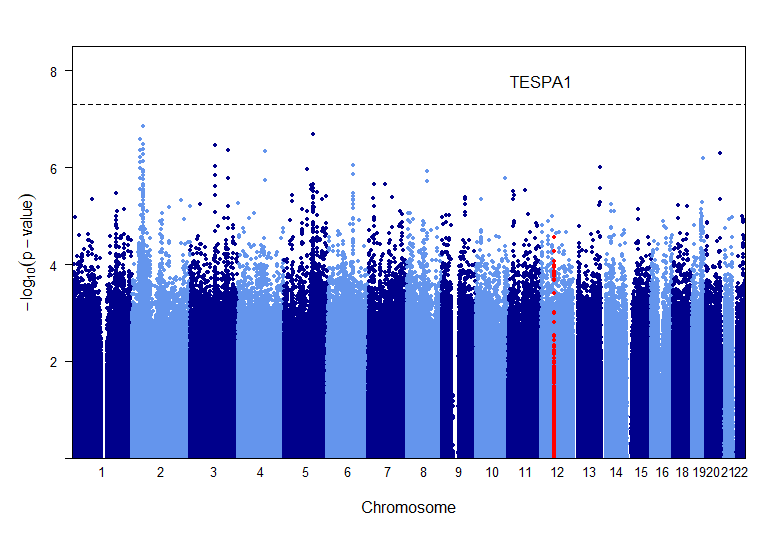
**

The plot shows P-values of all SNPs tested for association with epigenetic age acceleration. The y-axis presents log base 10 transformed P-value. The horizontal dashed line corresponds to the genome-wide association threshold (p=5 x 10^-8^). All SNPs in *TESPA1* were colored with red.

**Reference**

1. Destrieux C, Fischl B, Dale A, Halgren E. Automatic parcellation of human cortical gyri and sulci using standard anatomical nomenclature. *Neuroimage* 2010; **53**(1)**:** 1-15.

2. Sled JG, Zijdenbos AP, Evans AC. A nonparametric method for automatic correction of intensity nonuniformity in MRI data. *Ieee T Med Imaging* 1998; **17**(1)**:** 87-97.

3. Segonne F, Dale AM, Busa E, Glessner M, Salat D, Hahn HK *et al.* A hybrid approach to the skull stripping problem in MRI. *Neuroimage* 2004; **22**(3)**:** 1060-1075.

4. Navrady LB, Wolters MK, MacIntyre DJ, Clarke TK, Campbell AI, Murray AD *et al.* Cohort Profile: Stratifying Resilience and Depression Longitudinally (STRADL): a questionnaire follow-up of Generation Scotland: Scottish Family Health Study (GS:SFHS). *Int J Epidemiol* 2018; **47**(1)**:** 13-14g.

5. Smith BH, Campbell A, Linksted P, Fitzpatrick B, Jackson C, Kerr SM *et al.* Cohort Profile: Generation Scotland: Scottish Family Health Study (GS:SFHS). The study, its participants and their potential for genetic research on health and illness. *Int J Epidemiol* 2013; **42**(3)**:** 689-700.

6. Min JL, Hemani G, Smith GD, Relton C, Suderman M. Meffil: efficient normalization and analysis of very large DNA methylation datasets. *Bioinformatics* 2018; **34**(23)**:** 3983-3989.

7. Pidsley R, Wong CCY, Volta M, Lunnon K, Mill J, Schalkwyk LC. A data-driven approach to preprocessing Illumina 450K methylation array data. *Bmc Genomics* 2013; **14**.
